# Photosynthetic lesions can trigger accelerated senescence in *Arabidopsis thaliana*

**DOI:** 10.1093/jxb/erv393

**Published:** 2015-08-13

**Authors:** Jing Wang, Dario Leister, Cordelia Bolle

**Affiliations:** Ludwig-Maximilians-Universität München (LMU), Department Biologie I, Botanik, Großhaderner Str. 2–4, D-82152 Planegg-Martinsried, Germany

**Keywords:** *Arabidopsis*, photosynthesis, photosystem, ROS, senescence, STN7, STN8.

## Abstract

Senescence is a highly regulated process characterized by the active breakdown of cells, which ultimately leads to the death of plant organs or whole plants. In annual plants such as *Arabidopsis thaliana* senescence can be observed in each individual leaf. Whether deficiencies in photosynthesis promote the induction of senescence was investigated by monitoring chlorophyll degradation, photosynthetic parameters, and reactive oxygen species accumulation in photosynthetic mutants. Several mutations affecting components of the photosynthetic apparatus, including *psal-2*, *psan-2*, and *psbs*, were found to lead to premature or faster senescence, as did simultaneous inactivation of the STN7 and STN8 kinases. Premature senescence is apparently not directly linked to an overall reduction in photosynthesis but to perturbations in specific aspects of the process. Dark-induced senescence is accelerated in mutants affected in linear electron flow, especially *psad2-1*, *psan-2*, and *pete2-1*, as well as in *stn7* and *stn8* mutants and *STN7 and STN8* overexpressor lines. Interestingly, no direct link with ROS production could be observed.

## Highlight

While it is well known that the beginning of senescence is accompanied by the degradation of the photosynthetic machinery, photosynthesis may play a role in initiating senescence.

## Introduction

The last phase of the life cycle of annual plants like *Arabidopsis thaliana* is characterized by senescence, which usually coincides with the ripening of the seeds. Nevertheless, the onset of senescence can be observed in leaves at earlier stages of development. Leaf senescence may be interpreted as a response to various endogenous or environmental signals that are correlated with biological ageing. The environmental signals record cumulative exposure to stress resulting from abiotic influences such as extreme temperature, drought, nutrient limitation, shading, or oxidative stress precipitated by UV-B irradiation and ozone, and from biotic factors, chiefly infections by pathogens. The nutritional status of the plant can also play an important role in initiating senescence, because high concentrations of sugars reduce photosynthetic activity and induce leaf senescence (Jang *et al.*, 1997; Dai *et al.*, 1999; Quirino *et al.*, 2000; Moore *et al.*, 2003). In addition, detached leaves display strong symptoms of senescence during prolonged exposure to darkness or after treatment with ethylene (Weaver *et al.*, 1998). However, not only ethylene, but also hormones such as abscisic acid, jasmonic acid, and salicylic acid are involved in the senescence response of plants to their environment (Yang *et al.*, 2011; Arrom and Munne-Bosch, 2012; Fukao *et al.*, 2012; Oka *et al.*, 2012; Kim *et al.*, 2013).

Senescence is a tightly regulated degeneration process that is associated with dramatic changes in cellular metabolism and characterized by the dominance of catabolic over anabolic processes (Nam, 1997; van der Graaff *et al.*, 2006; Lim *et al.*, 2007; Wingler and Roitsch, 2008; Guiboileau *et al.*, 2010). These metabolic changes lead to yellowing of plant leaves, due to the progressive breakdown of chlorophylls and the recycling of most of their building blocks after redirection to other organs (Hurkman *et al.*, 1979). Death of leaf cells spreads in a highly controlled manner and finally encompasses the entire leaf or organism (Buchanan-Wollaston *et al.*, 2003). The degradation process begins in chloroplasts, and ultra-structural analysis has demonstrated that thylakoids experience sequential disassembly. First, the stroma-exposed thylakoid membranes disappear and the coherence of grana stacks is lost. There is then a massive increase in plastoglobuli, which are thought to contain the lipid-soluble products of thylakoid membrane degradation. The sequential dismantling of stroma and grana thylakoids implies that photosystem I (PSI) is degraded before photosystem II (PSII). However, reports on the sequence of degradation of the two photosystems during senescence are somewhat contradictory (Hilditch *et al.*, 1986; Yamazaki *et al.*, 2000; Humbeck and Krupinska, 2003; Tang *et al.*, 2005; Nath *et al.*, 2013). Loss of the cytochrome *b*_*6*_ *f* complex may precede the degradation of the photosystems and of ATP synthase, limiting electron transport between PSII and PSI (Holloway *et al.*, 1983; Guiamet *et al.*, 2002). This, together with the progressive loss of other proteins in the chloroplast, such as ribulose-1,5-bisphosphate carboxylase/oxygenase (Rubisco) and chlorophyll *a/b* binding proteins, results in a decrease in photosynthetic capacity (Hörtensteiner and Feller, 2002; Chrost *et al.*, 2007).

Several lines of evidence support a role for reactive oxygen species (ROS) in the cellular deterioration that ensues in response to various environmental stresses, and during natural senescence and dark-induced cell death processes (Guo and Crawford, 2005; Khanna-Chopra, 2012; Pinto-Marijuan and Munne-Bosch, 2014). ROS are by-products of photosynthesis and aerobic respiration (Navari-Izzo *et al.*, 1999). Superoxide (O_2_^−^) is generated in the Fe-S centres within PSI and diffuses to the membrane surface, where the enzyme superoxide dismutase catalyses its conversion into hydrogen peroxide (H_2_O_2_). H_2_O_2_ is then reduced to water by ascorbate with the aid of ascorbate peroxidases. In PSII, oxygen in the ground state (^3^O_2_) is excited to the singlet state (^1^O_2_) by the reaction-centre chlorophyll in the triplet excited state. If ^1^O_2_ is not quenched by β-carotenes or tocopherols it oxidizes proteins (especially those of PSII), unsaturated fatty acids, and DNA (Wagner *et al.*, 2004; Krieger-Liszkay *et al.*, 2008). Any disturbance in the photosynthetic equilibrium can therefore cause excess production of ROS. If stress conditions are prolonged, the level of ROS will eventually surpass the capacity of the detoxifying machinery, causing oxidative damage to cellular constituents—mostly to proteins, which are then degraded. Furthermore, the efficacy of the ROS detoxification system declines with age (Khanna-Chopra, 2012). Indeed, H_2_O_2_ levels increase dramatically in leaf tissue during senescence, but the compound is also considered to be a signal for the initiation of programmed cell death (Del Rio, 2015). In addition, exposing *A. thaliana* leaves to darkness for long periods activates a genetically controlled senescence programme. The transcriptome of leaves subjected to extended darkness contains a variety of signatures related to ROS-specific changes (Gadjev *et al.*, 2006). The transcriptome footprints of chloroplast-related ROS stress, on the other hand, decrease upon transfer into darkness, because the ROS-producing light reaction is no longer active (Rosenwasser *et al.*, 2011).

The association between ROS production and senescence is also evident in several mutants that display early cell death or senescence in parallel with symptoms of ROS stress. Examples include the pre-symptomatic leaves of the mutant *constitutive expression of PR genes5/onset of leaf death1 (cpr5/old1*), *jungbrunnen1 (jub1-1*), and lines that overexpress *ARABIDOPSIS A-FIFTEEN* (*AFF*) or *Cdf-RELATED GENE RESPONSIVE TO SENESCENCE* (*CRS*) (Miao *et al.*, 2004; Jing *et al.*, 2008; Chen *et al.*, 2012; Cui *et al.*, 2013). Woo *et al.* (2004) have reported that the gene encoding SENESCENCE-ASSOCIATED PROTEIN 1 is induced by oxidative stress, and that the delayed-senescence mutants *ore1*, *ore3*, and *ore9*, which exhibit enhanced leaf longevity, are more tolerant to oxidative stress in *Arabidopsis*. In mutants with reduced levels of intracellular H_2_O_2_, such as JUB1 overexpressor lines, *aff* and *crs*, senescence is also delayed (Chen *et al.*, 2012; Wu *et al.*, 2012; Cui *et al.*, 2013).

From these findings it can be concluded that photosynthesis could exert effects on senescence via two principal routes: (i) by influencing plant growth through the production of carbohydrates; and (ii) by producing excess ROS, in particular during light stress. A decrease in metabolism caused by impairments in photosynthesis should depress the developmental rate and, in consequence, delay senescence, while increased ROS accumulation should accelerate senescence induction. Therefore, a role for photosynthesis in senescence seems plausible, but its basic features are difficult to predict in detail. To address the questions of whether and how subtle impairments of the photosynthetic machinery might influence the onset of senescence, a set of mutations that affect the functions of the PSI complex, the intersystem electron transport, and the proteins involved in the regulation of photosynthesis has been investigated.

## Materials and methods

### Plant materials

The *A. thaliana* accessions Columbia (Col-0) and Wassilewskija-4 (WS) were obtained from the Nottingham *Arabidopsis* Stock Centre (NASC accession nos. N1092 and N2223) and served as wild types (WT). The mutant lines *psad2*-*1* (Ihnatowicz *et al.*, 2004), *psal*-2 (Pesaresi *et al.*, 2009*a*), *psbs* (Grasses *et al.*, 2002), *pete2-1* (Weigel *et al.*, 2003; Pesaresi *et al.*, 2009*b*), *stn7*-1 (Bonardi *et al.*, 2005), *stn8*-1 (Bonardi *et al.*, 2005), *stn7 stn8* (Bonardi *et al.*, 2005), the *STN7* overexpression line *oeSTN7* (Wunder *et al.*, 2013*a*), the *STN8* overexpression line *oeSTN8* (Wunder *et al.*, 2013*b*), *pam68l* (Armbruster *et al.*, 2013), *crr2*-2 (Hashimoto *et al.*, 2003), and *psae2*-1 (WS; Ihnatowicz *et al.*, 2007) have all been described previously. The *psan*-*2* allele was obtained from the Salk Collection (SALK_088053).

### Growth conditions

For seed production, plants were grown in the greenhouse under long-day conditions (16h light/8h dark) at a temperature of 19–22°C and exposed to light levels of about 200 µmol photons m^−2^s^−1^ light during the light phase.

For senescence experiments, plants were grown without fertilizer in a controlled environment (growth chamber) under long-day conditions (16h light/8h dark), but exposed to 100 µmol photons m^−2^s^−1^ light during the light phase, a relative humidity of 75%, and a temperature cycle of 22°C day/18°C night. Plants with different genotypes were always grown in parallel and in replicates.

Dark-induced senescence was imposed by transferring whole 30-day-old plants into the dark for 3, 7, or 10 days (during which plants were not watered) and returning them to the growth chamber at the indicated times.

### Measurement of leaf area

Whole plants were imaged non-invasively at 3 pm every 3 days from the emergence of leaf No. 6 at 20 days after seed germination (dag) until 35 dag. Pictures were taken with a digital camera. The leaf area was measured using the free software ImageJ (Staal *et al.*, 2004). Three biological replicates were used for each genotype and three independent measurements were performed in each case.

### Measurement of flowering time and leaf number at bolting

Flowering time (time to bolting) was defined as the number of days between germination and the appearance of a florescence stem of approximately 0.5cm. Leaf numbers were counted when the stem had reached a length of at least 2cm. Three independent measurements were performed on each of at least 10 biological replicates.

### Chlorophyll measurement

If not otherwise noted, chlorophyll levels were determined from 28 dag to 70 dag, and measurements were performed at 3 pm every 3 days. The mean of four readings with a portable Monitor chlorophyll meter (SPAD-502; Spectrum Technologies, Inc.) was obtained for each leaf from at least 20 individual plants. The meter measures the ratio vegetative index at 940nm and 650nm. Readings were taken at the midpoint of the leaf next to the main leaf vein. To verify the linear relationship between the SPAD values and chlorophyll content, leaves from Col-0 plants were extracted with 80% (v/v) aqueous acetone, and the absorbance of the extract at 663, 645, and 480nm was measured in a spectrophotometer. Fresh weights and chlorophyll concentrations were then determined according to Lichtenthaler *et al.* (2013). During natural senescence leaf No. 6 was measured, unless otherwise indicated. At least five biological replicates were used for each mutant and for Col-0. A *t*-test (two-sided, unequal variance) was performed to determine statistical significance of differences between WT and mutant samples.

### Chlorophyll fluorescence measurements

*In vivo* chlorophyll a fluorescence of single leaves was measured using the Dual-PAM 100 (Walz) according to Pesaresi *et al.* (2009*a*). The fluorescence of dark-adapted leaves (F_0_) was measured first, before they were exposed to pulses (0.5 s) of red light (5000 μmol m^−2^ s^−1^) to determine the maximum fluorescence (F_m_) and the maximum quantum yield (F_v_/F_m_, where F_V_ is the variable fluorescence [F_m_ − F_o_]) as a measure of the functionality of PSII. The light dependence of the photosynthetic parameters 1−qP and the effective quantum yield of PSII (ϕ_II_) were determined by applying increasing red light intensities (0–2000 μmol m^−2^ s^−1^) at 15-min intervals before the steady-state fluorescence (F_S_) was measured and a red light pulse (5000 μmol m^−2^ s^−1^) was applied to determine F_m_’. The relevant values were calculated using the following equations (Maxwell and Johnson, 2000): effective quantum yield of PSII, ϕ_II_ = (F_m_’ − F_s_)/F_m_, photochemical quenching, qP = [(F_m_’ − F_s_)/(F_m_’ − F_0_)], and non-photochemical quenching (NPQ), qN = [(F_m_ − F_m_’)/(F_m_ − F_0_)].

In all experiments, three plants were analysed for each genotype, and means and standard deviations were calculated; three independent experiments were performed in each case.

### H_2_O_2_ detection

Thirty-day-old leaves were vacuum-infiltrated in the dark for 5min with a 1mg/ml solution of 3, 3′-diaminobenzidine (DAB) in water brought to pH 3.8 with 0.1% HCl. After 8h in the dark at room temperature in the same solution, the leaves were illuminated for 15min (during which areas containing H_2_O_2_ turned brown), then washed twice in 80% ethanol at 80°C to remove chlorophyll and fixed in 70% glycerol. For each experiment, three plants of each genotype were analysed. Images were taken with a digital camera.

To investigate the kinetics of H_2_O_2_ production in chloroplasts, leaf protoplasts were exposed to 2′,7′-dichlorodihydrofluorescein diacetate (H_2_DCFDA). Enzymatic deacetylation and subsequent oxidation by H_2_O_2_ generate 2′,7′-dichlorofluorescein (DCF), which was quantified by fluorescence microscopy (Naydov *et al.*, 2012). Protoplasts were prepared from 30-day-old leaves (pre-senescence). To this end, the Tape-*Arabidopsis*-Sandwich method was used to isolate protoplasts of the different mutants (Wu *et al.*, 2009). Then, H_2_DCFDA (5 µM) was added to the washing buffer, and the protoplasts were incubated in the dark for 30min and washed twice. Protoplasts were always kept in darkness and resuspended in MMg solution (0.4M mannitol, 15mM MgCl_2_, 4mM MES pH 5.7). The protoplasts were viewed with a Fluorescence Axio Imager microscope (Zeiss) and protoplasts were located by their chlorophyll fluorescence. The increase in DCF fluorescence observed upon illumination via the 38GFP filter (Zeiss) was then monitored for 2min by collecting a timed series of images. The first picture taken immediately after illumination was defined as the ‘dark’ state. At least three chloroplasts each in a minimum of 10 protoplasts were followed and the intensity of fluorescence was measured using the Axiovision software (Zeiss). The mean level at 30 s after the onset of illumination was calculated from the linear portion of the curve.

### Immunodetection

Either 30- or 42-day-old leaves were ground in 2× SDS loading buffer to extract total protein. Proteins fractionated by SDS gel electrophoresis (12%) were subsequently transferred to polyvinylidene difluoride membranes. After blocking with TBST (10mM Tris pH 8.0, 150mM NaCl, and 0.1% Tween 20) supplemented with 5% (w/v) milk powder, the membranes were incubated with antibodies raised against subunits of PsaD (Agrisera), or the β-subunit of cpATPase (obtained from Jörg Meurer, University of Munich). After incubation with the appropriate secondary antibody, the signals were detected by enhanced chemiluminescence (ECL kit; Amersham Bioscience) using an ECL reader system (Fusion FX7; PeqLab) and quantified with Bioprofile software (PeqLab).

## Results

### Quantifying senescence in wild-type plants

Senescence is invariably accompanied by the degradation of chlorophyll. Therefore, chlorophyll content was non-invasively measured in WT and mutant plants, after establishing a linear relationship between conventional chlorophyll measurements and SPAD units (Supplementary Fig. S1A). To determine the timeline of age-dependent senescence, the chlorophyll contents of leaves No. 6 and 9 were measured over the course of each plant’s lifetime (Supplementary Fig. S1B). Because measurements of leaf No. 9 were only possible after 30 dag, leaf No. 6 was chosen to monitor the onset and progression of senescence. In WT plants grown under long-day conditions, net loss of chlorophyll in this leaf began at 33±2 dag.

To investigate the relationship between leaf development and chlorophyll accumulation, the rate of expansion of leaf No. 6 was also measured in Col-0 (Supplementary Fig. S1C). Leaf growth ceased around 30 dag, just 3 days before chlorophyll levels began to decline, and about 6 days before bolting (36.8±1.4 dag). Therefore, the best timeframe in which to track senescence on the basis of photosynthetic parameters was to begin measurements on leaf No. 6 just before 30 dag, prior to the cessation of vegetative growth and the subsequent onset of senescence. The total chlorophyll content of leaf No. 6 during the lifetime of WT plants (Col-0) was monitored in 10 separate experiments depicted in Supplementary Fig. S1D and the trend was consistent between the different repeats, indicating a high level of reproducibility.

### Photosynthetic mutants for senescence measurements

To test the effects of variations in the overall efficiency of photosynthesis on senescence, 13 mutants were selected with defects in proteins involved in the photosynthetic light reactions (Table 1). Four mutants were defective in PSI (*psad2-1* in the subunit PsaD, *psal-2* in PsaL, *psan-2* in PsaN, and *psae2-1* in PsaE), whereas the *pete2-1* mutant has reduced amounts of the intersystem electron transporter plastocyanin (PetE). Ccr2 and Pam68L are required for the formation of a functional NAD(P)H dehydrogenase (NDH) complex (Hashimoto *et al.*, 2003; Armbruster *et al.*, 2013); PsbS is required for NPQ (measured as qN) and has a photoprotective role under high light (Li *et al.*, 2000; Grasses *et al.*, 2002; Roach and Krieger-Liszkay, 2012). Furthermore, knockout mutants were studied, along with the mutants overexpressing the kinases STN7 and STN8, which are involved in photosynthetic acclimation (Bellafiore *et al.*, 2005; Bonardi *et al.*, 2005; Vainonen *et al.*, 2005; Pesaresi *et al.*, 2011; Wunder *et al.*, 2013*a*).

**Table 1.** List of mutants used in this work

Mutant	Gene	Protein/function	Complete loss of protein function
**PSI**			
*psad2-1*	*At1g03130*	D subunit of PSI	no
*psae2-1*	*At2g20260*	E2 subunit of PSI	no
*psal-2*	*At4g12800*	L subunit of PSI	yes
*psan-2*	*At5g64040*	N subunit of PSI	yes
**Intersystem electron transport**			
*pete2-1*	*At1g20340*	plastocyanin	no
**NDH complex**			
*crr2-2*	*At3g46790*	NDH assembly	yes
*pam68l*	*At5g52780*	NDH assembly	yes
**Protection and acclimation**			
*psbs-1*	*At1g44575*	dissipation of excess light energy, PsbS	yes
*stn7-1*	*At1g68830*	thylakoid protein kinase STN7	yes
*stn8-1*	*At5g01920*	thylakoid protein kinase STN8	yes
*stn7 stn8*	*At1g68830/ At5g01920*	double mutant of *stn7*-1 and *stn8*-*1*	yes
*oeSTN8*	*At5g01920*	overexpressor of STN8	-
*oeSTN7*	*At1g68830*	overexpressor of STN7	-

For references see ‘Material and methods’.

### General developmental behaviour of photosynthetic mutants

To assess the general developmental behaviour of the different mutants used in this study, their flowering time, maximum leaf size, and maximum chlorophyll content were studied. Two parameters were used to measure flowering time, day of bolting and leaf number at the time of bolting (Table 2). In comparison to WT plants, *psan-2* and *crr2*-2 mutants flowered about 3 and 6 days earlier, respectively. This effect was statistically significant (*P* < 0.05) and was also manifested by the lower number of rosette leaves at bolting time. The *stn8-1*, *oeSTN8*, and *oeSTN7* lines also flowered earlier than WT, but *oeSTN8* and *oeSTN7* displayed about same number of rosette leaves as WT at bolting time. All other mutants flowered at the same age as WT plants.

**Table 2.** Days to bolting and leaf number at bolting time in WT and mutant plants on soil in a growth chamber

Line	Days to bolting^a^ (dag)	Leaf number at flowering^b^ (dag)	Day of highest chlorophyll content (dag)	SPAD units on day of highest chlorophyll content	Difference between day of bolting and day of highest chlorophyll content	Number of days between maximum chlorophyll content and 50% reduction
WS	26.0±1.5	7.5±0.7	28	21.6	-2.0	15.6±0.8
*psae2-1* ^*c*^	28.0±1.5	6.0±0.5	27	20.0	1.0	13.7±1.0
Col-0	36.8±1.4	16.8±0.8	33	26.1	3.8	22.7±0.9
*psal-2*	34.8±3.2	15.3±0.8	33	27.4	1.8*	15.0±1.7*
*psan-2*	33.5±2.0*	14.6±1.3*	30	25.3	3.5	15.0±2.0*
*stn7 stn8*	35.5±2.0	15.8±0.7	33	25.8	2.5	16.6±1.4*
*psbs*	35,3±0.5	17.9±0.6	30	25.6	5.3	17.2±0.8*
*stn8-1*	32.8±1.8*	15.0±0.7*	30	26.4	2.8	18.8±1.8
*pam68l*	35.7±1.0	16.3±0.6	33	23.8	2.7	20.4±1.0
*oeSTN8*	32.3±1.1*	16.5±0.7	30	31.6	2.3	20.5±1.3
*psad2-1*	37.2±2.7	17.8±1.2	33	23.9	4.2	21.2±1.9
*crr2-2*	30.5±2.0*	15.3±0.8	30	22.1	0.5*	21.7±1.3
*pete2-1*	36.0±1.5	15.0±1.0	30	23.2	6.0*	22.8±1.2
*stn7-1*	36.9±1.5	15.8±0.8	33	25.7	3.9	23.6±1.3
*oeSTN7*	33.3±2.1*	17.3±0.6	29	31.0	4.3	24.6±1.5

^a^ Number of days from germination to the development of an inflorescence stem of approximately 0.5cm length.

^b^ Leaf number of the rosette was counted when shoot length was about 2cm. Standard error is given.

^c^ In WS background.

* Value is significantly different from the respective WT *(t*-test value *P* < 0.05).

### Timing of chlorophyll loss during age-dependent senescence

Because chlorophyll degradation is the first detectable symptom of senescence, the chlorophyll contents of the different photosynthesis mutants (Table 1) were compared during age-dependent senescence (Supplementary Fig. S2). The PSI mutants *psan-2* and *psal-2* showed early senescence, whereas *psad2-1*, *psae2-1* and the two NDH mutants, *crr2-2* and *pam68l*, behaved like their respective WT progenitors. The mutants *stn7-1* and *stn8-1* and the corresponding overexpression lines behaved like WT, but the double mutant displayed premature senescence.

The chlorophyll content of leaf No. 6 in mutants reached its maximum between day 29 and day 33 in Col-0 genotypes, and on day 27 or 28 in WS strains (Table 2), which confirms that not all mutants exhibit the same developmental time course. Notably in the early-flowering mutants *psan-2*, *crr2-2*, *stn8-1*, *oeSTN7*, and *oeSTN8*, chlorophyll content peaked earlier than in the corresponding WT (Table 2). In addition, chlorophyll levels in *psbs* and *pete2-1* peaked 3 days earlier than in Col-0. In the *psad2-1*, *pete2-1*, *pam68l*, and *crr2-2* strains, peak levels were also lower than in the WT controls. This finding can be partially explained as a direct effect of the photosynthetic impairment caused by the mutation, insofar that the genetic defect results in lower growth rates and reduced leaf area, especially in *crr2*-*2* plants. On the other hand, *oeSTN7* and *oeSTN8* plants actually accumulated more chlorophyll than WT.

**Figure 1.**
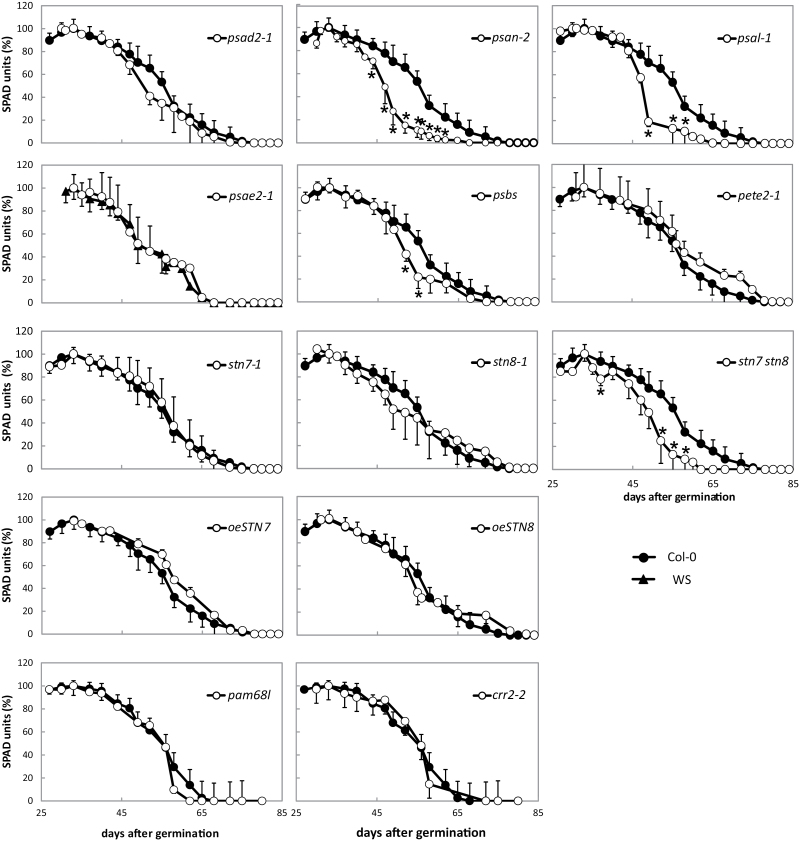
Time course of age-dependent senescence of Col-0 and mutants adjusted to the day on which the chlorophyll content reached its maximum. The chlorophyll content of leaf No. 6 was measured. The highest chlorophyll content was set to 100% and the day on which the chlorophyll content peaked was aligned with that for Col-0 or WS as appropriate. Days after germination (dag) refers to the respective WT. Error bars (for visual clarity only in one direction) represent the SE (*n* = 4–6 independent experiments with three plants each). Significant deviation from WT (*P* < 0.05) is marked with an asterisk.

To compensate for these variations in developmental rates, the points of highest chlorophyll content were aligned with those in Col-0 (Fig. 1). This plot clearly reveals that chlorophyll content declines more rapidly in the mutant lines *psan-2*, *psal-2*, *psbs*, and *stn7 stn8* than in Col-0. This is also obvious from Table 2, in which the length of time needed for chlorophyll levels to fall by 50% from their respective peak is listed. Again, only the double mutant *stn7 stn8*, but not the parental single mutant lines, showed this behaviour. No deviation from the WT was observed in the STN7 and STN8 overexpressor lines, although they flowered earlier.

The *psan-2* mutant not only flowered 3 days earlier than WT, it also entered senescence 3 days prior to Col-0. The *psal-2* mutant showed no significant shift in flowering date or onset of senescence, but a strong decline in chlorophyll content after day 45 was observed. This faster decline was also seen in *psbs* and the *stn7stn8* double mutant. Thus, overall, only a subset of the mutations examined resulted in a change in senescence behaviour, leading to premature or more rapid senescence. This clearly indicates that certain perturbations of photosynthetic efficiency can specifically influence the timing and rate of senescence.

### Changes in photosynthetic parameters during age-dependent senescence

**Figure 2.**
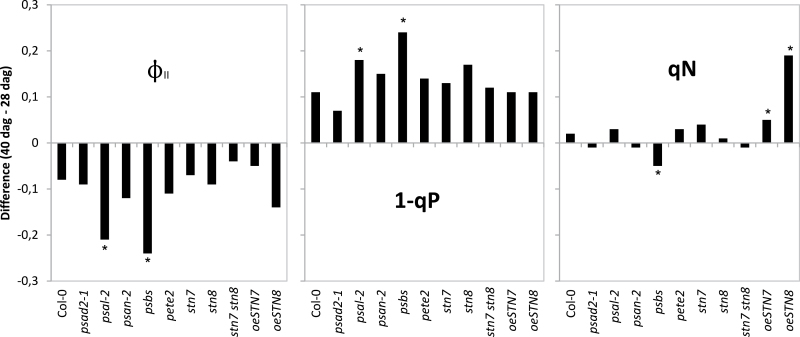
Effects of age-dependent senescence on photosynthetic efficiency in WT and mutant strains of *Arabidopsis thaliana* between 28 and 40 dag. The plots show the difference between the parameter values on 40 and 28 dags. Measured parameters were ϕ_II_, 1-qP, qN, and F_v_/F_m_. Significant differences from Col-0 are marked with an asterisk.

To further characterize the effects of age-dependent senescence on photosynthesis, the chlorophyll fluorescence parameters ϕ_II_, 1−qP, qN, and F_v_/F_m_ were measured for WT and those mutant plants that showed deviations from WT in the kinetics of senescence-dependent chlorophyll reduction at the onset of age-dependent senescence (Supplementary Tables S1 and S2, Fig. 2).

To assess photosynthetic status in the pre-senescent phase, fluorescence parameters were first measured at 28 dag. PSII was not affected in the mutants, as indicated by the F_v_/F_m_ values. While the effective quantum yield of PSII (ϕ_II_) is the fraction of absorbed energy utilized in both photosystems, 1−qP reflects the reduction state of the plastoquinone pool. ϕ_II_ values for *pete2-1* and *stn7 stn8* plants were significantly reduced compared to Col-0, and their 1−qP values also deviated significantly from Col-0, confirming previous results (Supplementary Table S1; Bonardi *et al.*, 2005; Pesaresi *et al.*, 2009*a*). The chlorophyll fluorescence parameter qN can be used to quantify NPQ, which is a measure of the fraction of energy that is dissipated as heat. A strong decline in qN was observed in *pete2-1* (attributable to the lower ΔpH resulting from reduced linear electron transport) and in *psbs* (Li *et al.*, 2000).

At 40 dag, Col-0 showed reduced ϕ_II_ and increased 1−qP values, but no significant change in F_v_/F_m_ and qN values compared to 28 dag. This suggests that PSII functioning and NPQ were still normal at this point, while the linear electron flow ‘downstream’ of PSII was already affected by senescence (Supplementary Tables S1 and S2, Supplementary Fig. S3). These differences become clear when the ratios between the photosynthetic parameters at 40 dag are compared to those at 28 dag (Fig. 2). This analysis identified *psal*-*2* (ϕ_II,_ 1−qP), *oeSTN8* (qN), and *psbs* (ϕ_II_, 1−qP and qN) as the genotypes with altered age-dependent changes in photosynthetic parameters. The *pete2-1* line also exhibited reduced qN and ϕ_II_ compared to Col-0, albeit to the same degree at both developmental stages, which argues that this effect is not related to senescence. F_v_/F_m_ was unaffected in all lines.

The ϕ_II_ and 1−qP values of the different lines at different ages were also plotted as a function of chlorophyll content (Supplementary Fig. S4). This graph demonstrates that chlorophyll amount and linear electron flow were directly correlated during senescence. On the other hand, no correlation between qN and chlorophyll content was observed, suggesting that additional factors play a role in mediating age-dependent changes in NPQ.

Because early ATPase depletion might play a role in the dramatic increase in NPQ observed in *oeSTN8*, the levels of ATPase were tested at various time points in this genotype. If ATPase is degraded before the photosynthetic apparatus, an increase in the pH in the lumen would be expected, which would account for the higher qN observed in *oeSTN8*. Indeed, amounts of the β subunit of the plastidic ATPase fell slightly between days 30 and 42 in *oeSTN8*, but not in Col-0, while levels of PsaD remained stable (Supplementary Fig. S5). Whether the ATPase is additionally differentially regulated during senescence cannot be ruled out at this point.

### ROS production in photosynthetic mutants

**Figure 3.**
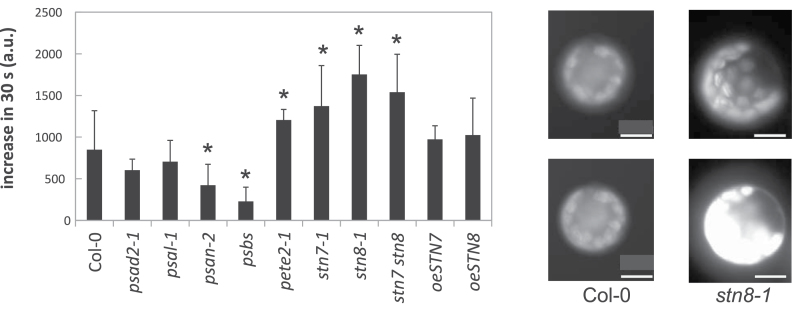
Detection of ROS by quantification of DCF in chloroplasts. The rise in fluorescence during the first 30 s of excitation was calculated. Significant deviation from WT (*P* < 0.05) is marked with an asterisk. Representative protoplasts imaged by fluorescence microscopy via the GFP filter at the beginning of the experiment and 1min later are shown (left: Col-0; right: *stn8-1*). Bar = 20 µm.

To test whether ROS levels were increased in the photosynthetic mutants, protoplasts isolated from WT and mutants were incubated with the cell-permeable fluorescein precursor H_2_DCFDA. Intercellular esterases can cleave the acetate groups and oxidation converts the component into the highly fluorescent DCF, which is retained in the cell. DCF was mainly observed in the chloroplasts and the fluorescence was quantified over a period of 2min of illumination at DCF’s excitation wavelength (495nm). Figure 3 shows the rate of increase of DCF over 30 s. In this assay *stn7-1*, *stn8-1*, the double mutant, and *pete2-1* displayed higher values compared to Col-0, whereas the values for *psbs* and *psan-2* were lower, suggesting that ROS production was attenuated in the latter genotypes. At first sight, this seems counter-intuitive, because more excitation pressure (like in *psbs*, see Fig. 2) should lead to higher ROS and trigger senescence. In fact, *psbs* displayed higher excitation pressure at accelerated senescence but less ROS production, suggesting that at least in some instances increased ROS levels and accelerated senescence do not correlate, possible owing to compensation in such genotypes.

### Loss of chlorophyll during dark-induced senescence

**Figure 4.**
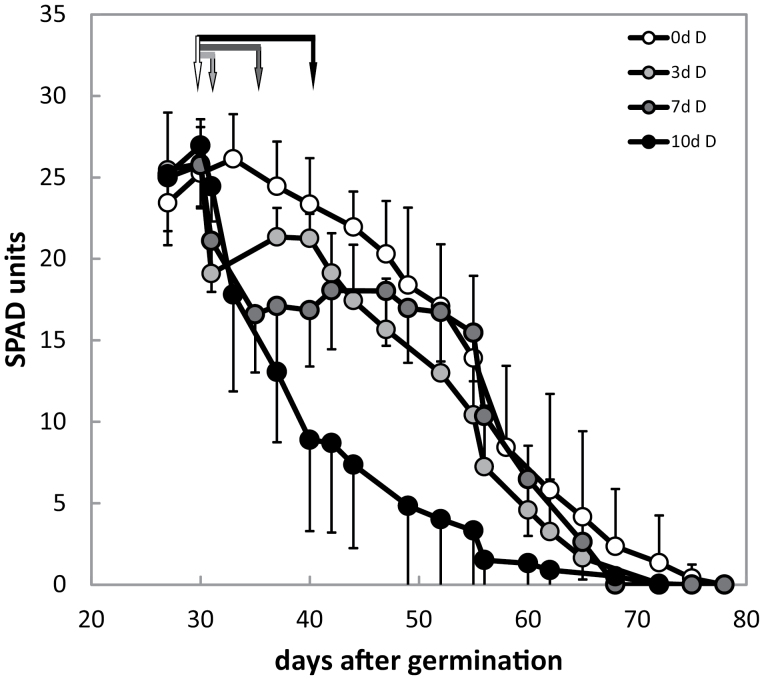
Dark-induced senescence in Col-0. Col-0 plants were kept in the dark for 3, 7, and 10 days and returned to light after the indicated time. Arrows indicate the respective endpoint of the dark period. Error bars represent the SE (*n* = 12 measurements from four different experiments) and in some cases are only indicated in one direction to avoid overlaps.

Leaf senescence occurs as the result of natural aging, but it can also be induced artificially by stresses, such as by shading individual leaves (Weaver and Amasino, 2001; Keech *et al.*, 2007). Dark-induced senescence allows one to bypass the generalized effects on development seen in some mutants, which are expected to have an impact on age-dependent senescence. Simply covering whole plants evokes dark-induced senescence, and this treatment was applied to 30-day-old plants for 3, 7, or 10 days. The chlorophyll content quickly declined in the dark, but recovered when WT plants that had been kept in darkness for 3 or 7 days were returned to normal long-day conditions (Fig. 4). However, WT plants that had spent 10 days in the dark did not recover and early senescence was induced. This suggests that there is a ‘point of no return’ that separates the pre-senescence stage from the phase of irreversible senescence. In fact, in several plant species, dark-induced senescence is fully reversible upon re-illumination and the leaves can re-green, but the re-greening ability depends on the duration of incubation in the dark (Parlitz *et al.*, 2011). It has also been shown that in dark-treated plants the number of chloroplasts is reduced by one-third after 6 days, which could also explain the reduction in chlorophyll observed after prolonged incubation in the dark (Keech *et al.*, 2007).

**Figure 5.**
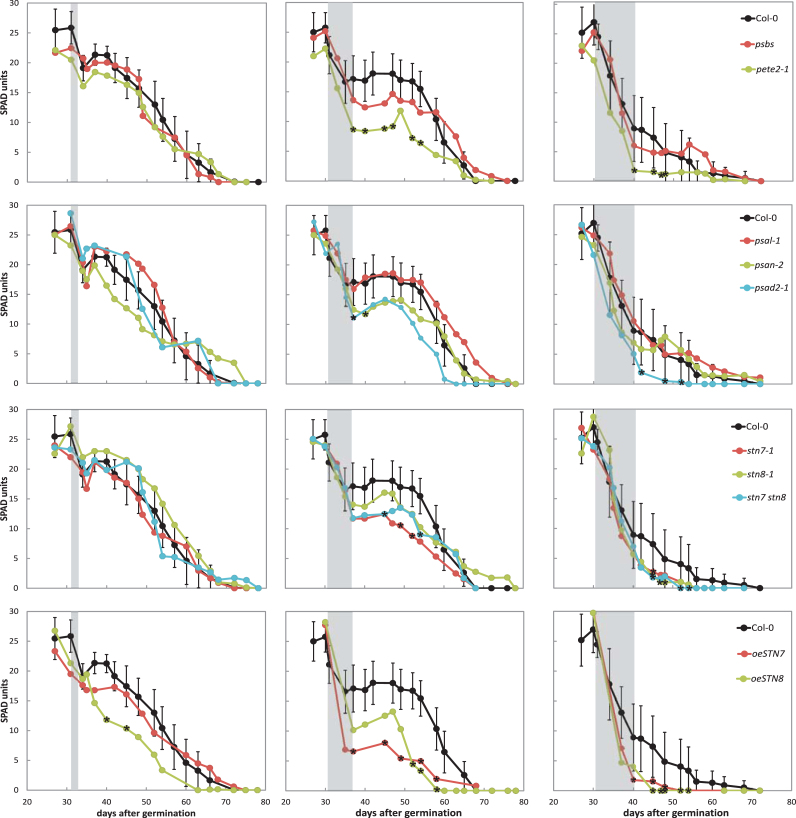
Levels of chlorophyll in leaf No. 6 of Col-0 and mutant plants kept for 3 (left), 7 (middle), and 10 (right) days in the dark. Shaded areas indicate the respective dark period. Error bars represent the SE (*n* = 12 measurements from four different experiments) and are only indicated in WT to avoid overlaps. Significant deviation from WT (*P* < 0.05) is marked with an asterisk.

As shown in Fig. 5, after 3 days of darkness, all loss-of-function mutants exhibited essentially the same decrease in chlorophyll content as WT. However, *oeSTN8* showed an earlier decline following transfer into the light. Indeed, no real recovery phase in which chlorophyll levels increased was observed in *oeSTN7* or *oeSTN8* plants.

After 7 days of darkness, the chlorophyll level had significantly decreased to 25–50% of the starting value in the mutants *psan-2*, *psad2-1*, *pete2-1*, and *oeSTN7*, whereas WT chlorophyll levels fell by one-third (Fig. 5). Nevertheless, all loss-of-function mutants recovered to some extent, albeit usually at a slower rate than WT. WT plants lost about 67% of their chlorophyll after 10 days in the dark (Fig. 5). However, in *pete2-1*, *psad2-1*, *stn7-1*, *stn8-1*, *stn7 stn8*, *oeSTN7*, and *oeSTN8*, the chlorophyll content declined more rapidly and led to early plant death. For *psan-2* and *psbs* some recovery was observed even after 10 days of darkness, leading to a delay in the onset of senescence. The *crr2-2* and *pam68l* lines behaved like WT under all three conditions (data not shown).

### ROS production after dark-induced senescence

**Figure 6.**
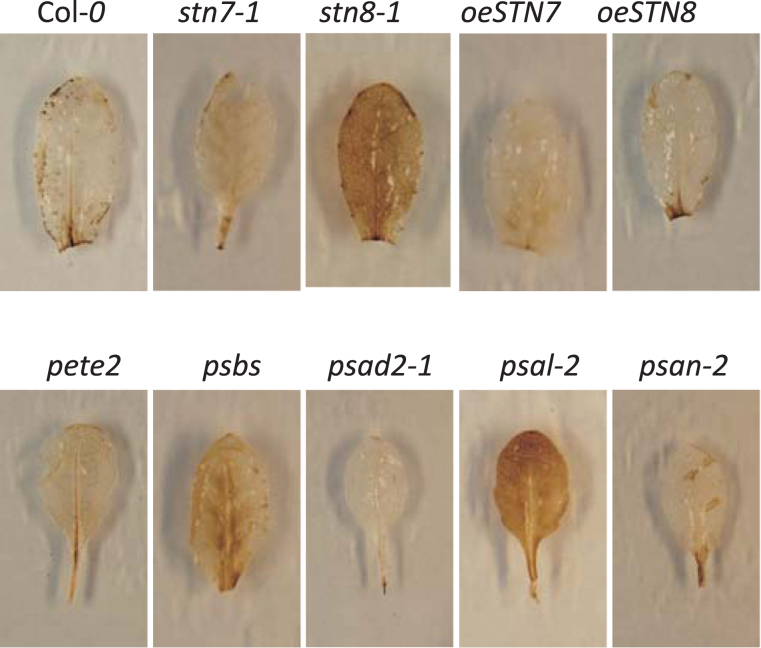
Detection of H_2_O_2_ using DAB staining in leaves of Col-0 and mutants at 40 dag. The intensity of the brown stain reflects the level of ROS accumulation.

To test whether the ROS levels increased upon re-illumination after incubation in the dark, staining with DAB, which has been shown to stain H_2_O_2_ in mature *Arabidopsis* rosette leaves, was performed (Bindschedler *et al.*, 2006; Daudi *et al.*, 2012). To this end, leaves were harvested from plants kept in the dark for 7 days, and incubated in the light while staining with DAB. The intensity of the brown colour reflects the amount of H_2_O_2_, and in *psal-2*, *psbs*, *stn8-1*, and—to a lesser extent—in *pete2-1*, *stn7-1*, and *oeSTN7*, staining was stronger than in Col-0, indicating that these mutants accumulated more H_2_O_2_ after re-illumination (Fig. 6). This could be due to inefficiency of the photosynthetic apparatus in these lines, or might result from more indirect effects.

## Discussion

Leaf senescence represents an important phase in the plant life cycle (Nam, 1997; Buchanan-Wollaston *et al.*, 2003; Lim and Nam, 2005). It is a highly regulated degeneration process, during which leaf cells undergo a defined sequence of changes in structure, metabolism, and gene expression. Chloroplast breakdown is the earliest event, and the most significant because chloroplasts contain up to 70% of total leaf protein. At the metabolic level, catabolism of chlorophyll, membrane lipids, and macromolecules, including proteins and RNA, slowly replaces carbon assimilation.

Although it has been reported that senescence alters the function and composition of the photosynthetic complexes in association with changes in the total soluble protein content (Nath *et al.*, 2013), whether the reverse is true—i.e. whether perturbations of photosynthesis affect the timing of senescence—has received little attention so far.

### Acceleration of age-dependent senescence can be linked to a faster life cycle, a higher 1−qP, or a lower effective quantum yield of PSII

In most of the photosynthetically compromised mutants examined here, the timing of senescence onset was essentially unaffected. However, in several of them (*psan-2*, *psbs*, *stn8-1*, *oeSTN7*, *oeSTN8*, *crr2-2*, and *pete2*-*1*) chlorophyll levels peaked 3–4 days earlier than in Col-0. This suggests that early age-dependent senescence might in part be attributable to a general acceleration in the pace of development. This is supported by the fact that all but two of these mutants (*psbs* and *pete2-1*) also flowered earlier. Once chlorophyll levels went into decline, rates of senescence in *psal-2*, *psan-2*, *stn7 stn8*, and *psbs* were also higher than in WT, although only *psan-2* showed the early flowering indicative of a generalized increase in rate of progression through the life cycle. Some photosynthetic mutants are severely stunted and have pale yellow leaves, suggesting that, in these cases, the alterations in energy metabolism have a severe impact on development. For this reason, attention was focused in this study on mutants that showed only mild impairment of general development. Among these, only *psan-2* and *psbs* also showed early onset of age-dependent senescence.

Interestingly, F_v_/F_m_ remained unchanged in all cases, even in the later stages of senescence, suggesting that PSII centres are still intact. Analyses of fluorescence quenching parameters in Col-0 at steady-state photosynthesis showed that 1−qP was slightly increased during senescence, whereas the ϕ_II_ value slightly decreased (Supplementary Tables S1 and S2). This most probably reflects the fact that the different thylakoid complexes are degraded at different rates during senescence, which leads to imbalances in electron flow and proton gradient formation that become manifest in these parameters. Nevertheless, no significant changes in PSI composition (measured as PsaD) or ATPase were observed in Col-0 between days 30 and 42, which is consistent with the analysis by Nath *et al.* (2013) that showed that the PSI reaction centre and ATPase were stable at later stages of senescence.

In *psal-2* and *psbs* a decline in ϕ_II_ and an increase in 1−qP were noted, whereas these effects were absent in the other two mutants that exhibited premature age-dependent senescence (*psan-2* and *stn7 stn8*) (Supplementary Table S2). In *stn7 stn8*, a reduction in ϕ_II_ and an increase in 1−qP were already obvious on day 28, suggesting that at this time the double mutant was already subject to senescence. The parameters ϕ_II_ and 1−qP showed a higher correlation with the chlorophyll content (Supplementary Fig. S4), implying that these parameters might also correlate in some cases with early onset of senescence.

Values of qN did not change much during ageing in Col-0, but two of the mutants, namely *psbs* and *oeSTN8*, did not conform to this pattern. In *psbs* plants, ϕ_II_ and qN were strongly reduced at 40 dag, whereas 1−qP was higher, indicating drastic changes in the thylakoid redox state and NPQ. Li *et al.* (2000) have shown that a reduced qN value is a general feature of mutants lacking PsbS (and was also observed in the *psbs* mutant in this study). Therefore, a reduced NPQ could be involved in preventing premature cell death. Conversely, in *oeSTN8*, the qN value at 40 dag was increased by 2-fold compared to that at 28 dag, but this was not reflected by a delay in senescence, indicating that NPQ *per se* does not directly correlate with senescence. The increased qN values seen in *oeSTN8* might reflect an earlier onset of plastidic ATPase degradation in the lines, which results in a higher transmembrane proton gradient (see Supplementary Fig. S5).

The results reveal that only specific perturbations of the photosynthetic machinery seem to lead to an accelerated senescence and programmed cell death. This certainly relates to the fact that photosynthesis mutants with extreme developmental impairments were excluded from this study. However, this observation also suggests that only very specific imbalances in photosynthesis can trigger early senescence.

### Accelerated senescence by dark-incubation is dependent on the presence of PsaD, PsaN, PetE, STN7, and STN8

To mitigate effects due to differences in developmental rates between mutants and fluctuations in environmental factors in the climate chambers, senescence was induced in leaves from Col-0 and photosynthetic mutants by incubating whole plants in darkness. That senescence can be induced by exposing plants to continuous darkness has been known for many years, and many studies of this ‘dark-induced’ senescence have been published. It was shown previously that senescence is induced in detached *Arabidopsis* leaves in response to darkness (Fujiki *et al.*, 2001; Guo and Crawford, 2005). However, relatively few studies have used adult leaves to probe dark-induced senescence, in *Arabidopsis* or any other species (van der Graaff *et al.*, 2006; Parlitz *et al.*, 2011), and the few such reports have been somewhat contradictory in their conclusions. Therefore, this study focused on studying the senescence of attached leaves in completely shaded plants.

In the present experiments, darkness was maintained for 3, 7, and 10 days. *Arabidopsis* Col-0 was able to resume growth even after 7 days in the dark, and total chlorophyll levels recovered after WT plants were returned to light. Many reports have shown that transfer of whole plants to darkness induces senescence in true leaves, and reversibility of senescence has also been observed, suggesting that chlorophyll and protein losses can reversed over the course of several days by returning plants to the light (Blank and McKeon, 1991; Kleber-Janke and Krupinska, 1997).

In several of the photosynthesis mutants analysed, namely *psad2-1*, *psan-2*, *pete2-1*, the *stn* mutants and overexpressor lines, extended incubation in darkness accelerated the onset of senescence upon subsequent exposure to light. PsaD, PsaN, and PetE are all important for the electron transport from the cytochrome *b*_*6*_ *f* complex to ferredoxin (Haldrup *et al.*, 1999; Ihnatowicz *et al.*, 2004; Pesaresi *et al.*, 2009*b*). The *psal-2* and *psbs* mutants, which are subject to premature age-dependent senescence, showed no early onset of senescence after dark stress, suggesting that different mechanisms were at work in these developmental programmes.

The primary function of STN7 is the phosphorylation of LHCII, which enables the latter to migrate to PSI and initiates a state transition, whereas STN8 phosphorylates PSII core proteins (Bellafiore *et al.*, 2005; Bonardi *et al.*, 2005; Vainonen *et al.*, 2005; Pesaresi *et al.*, 2011). Thus, these results suggest that prolonged exposure to darkness and acclimation to changing light conditions might trigger similar responses. Interestingly, both loss-of-function lines and overexpression lines of STN7 and STN8 are characterized by early onset of senescence, suggesting that any perturbation in these acclimation processes is detrimental to adaptation and induces early senescence.

A mutant called *thylakoid formation 1* (*thf1*) has recently been isolated, in which PSII–LHCII dynamics during dark-induced senescence are altered. THF1 interacts with LHCII proteins, suggesting that it is involved in a process similar to that mediated by the STN proteins. The selective stabilization of PSII–LHCII but not PSI in the *thf1* mutant during extended darkness accelerates senescence onset (Huang *et al.*, 2013).

### A combination of factors is needed to induce photosynthesis-triggered senescence

One factor that could drive early senescence is the accumulation of ROS. ROS are known to damage cell components and to be involved in senescence (Khanna-Chopra, 2012; Sabater and Martin, 2013). Chloroplasts, with their strong photo-oxidative potential, produce significant amounts of ROS. H_2_O_2_ is produced at PSI in particular, and is detoxified by several scavenging systems. The impairments in photosynthesis in the mutants used for this study could lead to an increase in ROS to levels that exceed the capacity of the scavenger system, leading to damage and eventually cell death. Indeed, for *pete2-1*, *stn7-1*, *stn8-1*, and *stn7 stn8* (age-dependent senescence) and also for *stn8-1*, *psbs*, *and psal-2*, accumulation of ROS (after dark-induced senescence) was higher than in WT. Nevertheless, strong effects in dark-induced senescence (see Fig. 5) did not correlate well with increased ROS accumulation. This is in contrast to Sabater and Martin (2013), who discuss that over-reduction of components of the photosynthetic electron transport cause increased ROS accumulation. Because the experiments described in the study of Sabater and Martin (2013) were performed in strong light conditions, this effect on ROS production should be dependent on the light intensity. In conclusion, at lower light intensities, as used here in this study, higher ROS levels played a minor role in inducing senescence.

None of the factors studied here—developmental effects influenced by reduced photosynthesis, photosynthetic parameters, and ROS accumulation—were clearly correlated with early senescence, indicating that several photosynthesis-associated factors might act in concert. The best correlations were found between dark-induced senescence and accumulation of ROS in lines with imbalances in phosphorylation of the photosystems. A reduction in the effective quantum yield of PSII (ϕ_II_) and increase of 1−qP was partially correlated with early age-dependent senescence, suggesting that over-reduction of the intersystem electron chain can trigger senescence. Interestingly, neither mutant in the NDH complex showed an altered senescence pattern under the conditions tested, suggesting that this complex does not play a major role in delaying senescence under the conditions used. This does not exclude that the NDH complex could play a role in senescence in other conditions and species as Zapata *et al.* (2005) have shown that in *Nicotiana tabacum* the loss of *ndhF* delays senescence.

Although it seems counter-intuitive that mutants affected in photosynthesis contribute to dark-induced senescence, some such effects were observed in this study. For three of the mutants, *pete2*, *psad2-1*, and *oeSTN7*, chlorophyll content was significantly reduced at the end of the dark period, suggesting that these mutants cannot cope with this stress. By contrast, the chlorophyll level in *oeSTN8*, *stn7-1*, *stn8-1*, and *stn7 stn8* started to decline significantly after returning to the light, suggesting that the lesions in the genotypes makes them more susceptible to stress related to re-establishing photosynthesis.

## Supplementary data

Supplementary material are available at *JXB* online.

Supplementary Figure S1. Establishment of a senescence assay.

Supplementary Figure S2. Age-dependent senescence of Col-0 and photosynthetic mutants.

Supplementary Figure S3. Changes in photosynthetic parameters during age-dependent senescence in Col-0.

Supplementary Figure S4. Scatter plot of chlorophyll data against different photosynthetic parameters.

Supplementary Figure S5. Age-dependent accumulation of the β-subunit of the cpATPase and PsaD in *oeSTN8* and Col-0.

Supplementary Table S1. Photosynthetic efficiency measured as F_v_/F_m_, ϕ_II_, 1−qP, and qN in leaf No.6 of WT and mutants of *Arabidopsis thaliana* at the beginning of age-dependent senescence (day 28).

Supplementary Table S2. Photosynthetic efficiency measured as F_v_/F_m_, ϕ_II_, 1−qP, and qN in leaf No.6 of WT and mutants of *Arabidopsis thaliana* at day 40.

Supplementary Data
