# Supplementary material for: Photosynthetic lesions can trigger accelerated senescence in Arabidopsis thaliana
Source: J Exp Bot. 2015 Aug 13;66(21):6891–903. doi: 10.1093/jxb/erv393 (PMC4623695; doi:10.1093/jxb/erv393)
Supplement: Supplementary Data [file supp_66_21_6891__index.html]

Photosynthetic lesions can trigger accelerated senescence in Arabidopsis thaliana — Supplementary Data 

# Photosynthetic lesions can trigger accelerated senescence in *Arabidopsis thaliana*

## Supplementary Data

Data files

- Supplementary Data - Supplementary Data
