# Supplementary material for: Photosynthetic lesions can trigger accelerated senescence in Arabidopsis thaliana
Source: J Exp Bot. 2015 Aug 13;66(21):6891–903. doi: 10.1093/jxb/erv393 (PMC4623695; doi:10.1093/jxb/erv393)
Supplement: Supplementary Data [file supp_erv393_Supplementary_Data_6_8_15.pdf]

Supplementary Data

Figure S1. Establishment of a senescence assay

A) Calibration of SPAD-502 readings with total leaf chlorophyll concentration in Col-0. Leaves of different ages were collected from mature plants grown in the greenhouse. The regression curve indicates a linear relationship between the two measurements. Chlorophyll content is plotted in µg/mg fresh weight (FW). The correlation coefficient (R^2) is given.

B) Comparison of the chlorophyll contents of leaves 6 and 9 during the development of *Arabidopsis thaliana* Col-0 under long-day conditions.

C) Area of leaf No. 6 during development of Col-0 and the early-flowering mutants *psan-2* and *stn8-1*. Error bars represent S.E. (*n* = 8 measurements). Slight reductions in leaf size during later stages are due to the effects of curling.

D) Chlorophyll content of leaf No. 6 of *Arabidopsis thaliana* Col-0 during age-dependent senescence. Measurements were performed on biological and experimental replicates and the mean value was calculated.

Figure S2. Age-dependent senescence of Col-0 and photosynthetic mutants. The chlorophyll content of leaf No. 6 was measured. Error bars represent the S.E. (*n* = 4–6 independent experiments with three plants each) and are only indicated on the WT plot to avoid overlaps.

Figure S3. Changes in photosynthetic parameters during age-dependent senescence in Col-0. Error bars represent the S.E. At 53 days only surviving leaves were included in the measurement.

Figure S4. Scatter plot of chlorophyll data against different photosynthetic parameters. An exponential trend line was added. Values for different lines (WT and mutants) and stages (28 dag, 40 dag and 53 dag) were combined. Data are from Fig. S4 and Supplementary Tables 1 and 2. The correlation coefficients (R^2) are given.

Figure S5. Age-dependent accumulation of the β-subunit of the cpATPase and PsaD in *oeSTN8* and Col-0. Leaves were harvested after either 30 days (1) or 42 days (2). Bands were quantified and the change relative to Col-0 at 30 days is given.

Supplementary Table 1. Photosynthetic efficiency measured as F_v/F_m, ϕ_{II}, 1-qP and qN in leaf No.6 of WT and mutants of *Arabidopsis thaliana* at the beginning of age-dependent senescence (day 28).

Supplementary Table 2. Photosynthetic efficiency measured as F_v/F_m, ϕ_{II}, 1-qP and qN in leaf No.6 of WT and mutants of *Arabidopsis thaliana* at day 40.

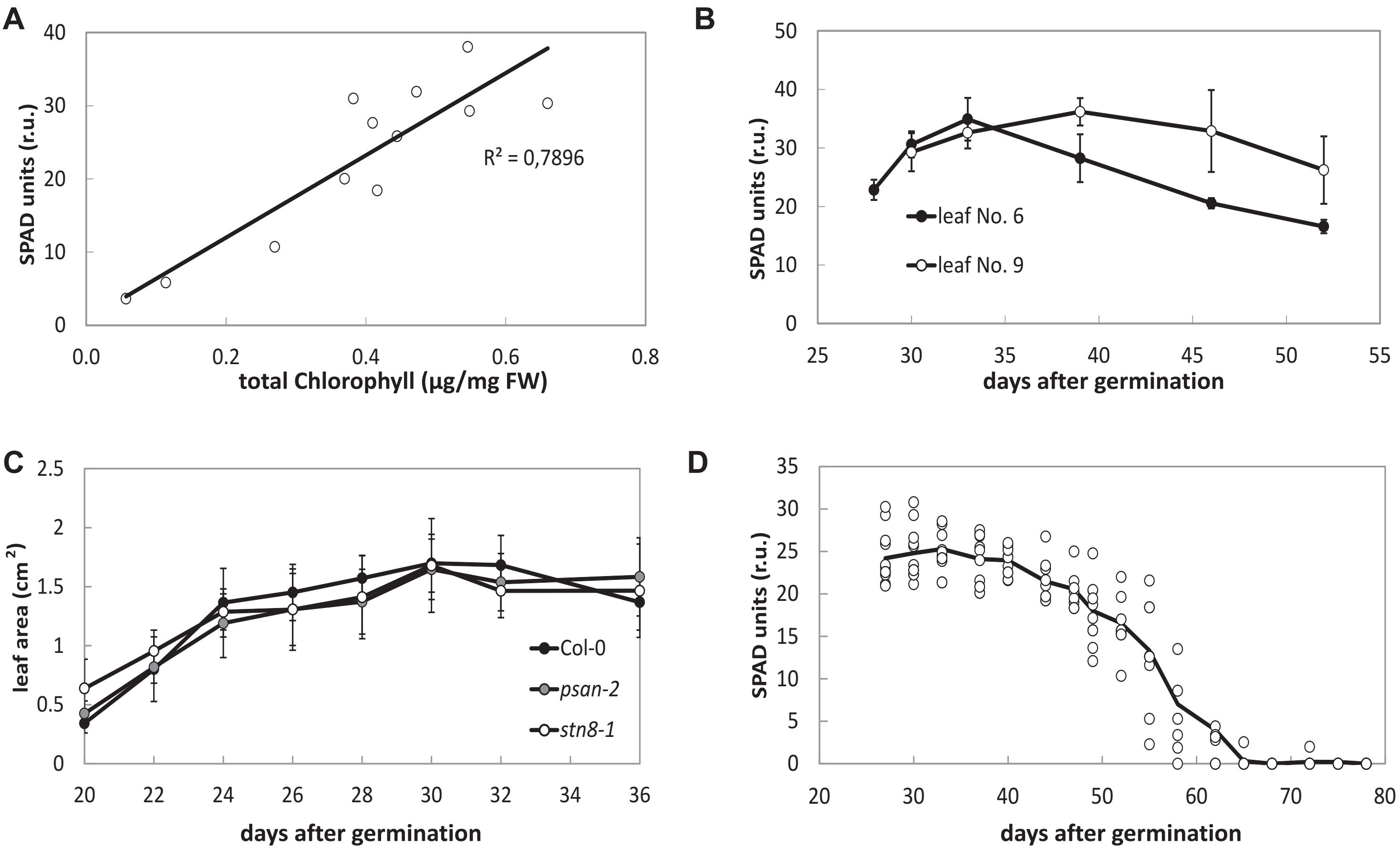

A
SPAD units (r.u.)
total Chlorophyll (µg/mg FW)
R² = 0,7896
B
SPAD units (r.u.)
days after germination
leaf No. 6
leaf No. 9
C
leaf area (cm²)
days after germination
Col-0
psan-2
stn8-1
D
SPAD units (r.u.)
days after germination

Figure S1

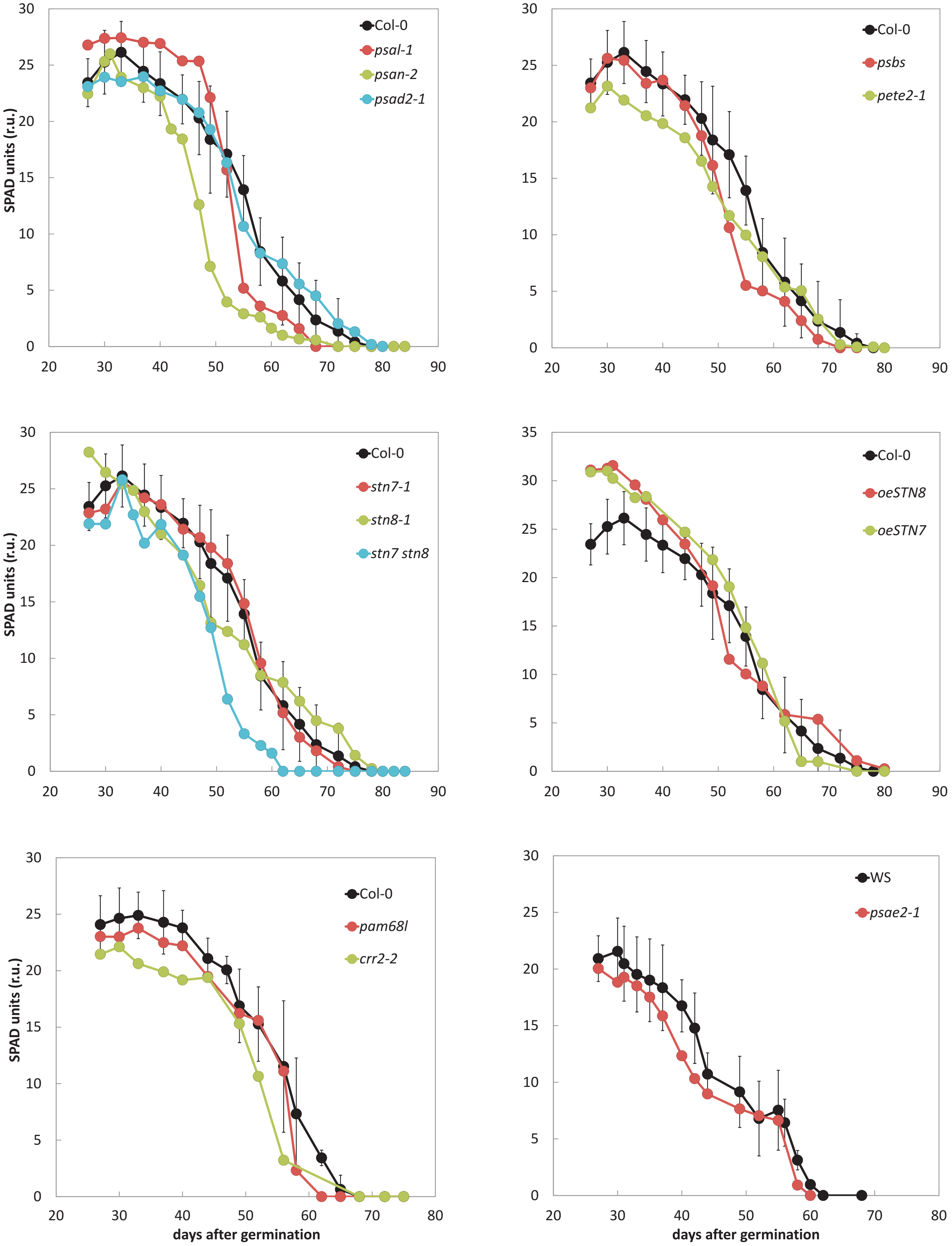

SPAD units (r.u.)
days after germination
Col-0
psal-1
psan-2
psad2-1
psbs
pete2-1
stn7-1
stn8-1
stn7 stn8
oeSTN8
oeSTN7
pam68l
crr2-2
WS
psae2-1

Figure S2

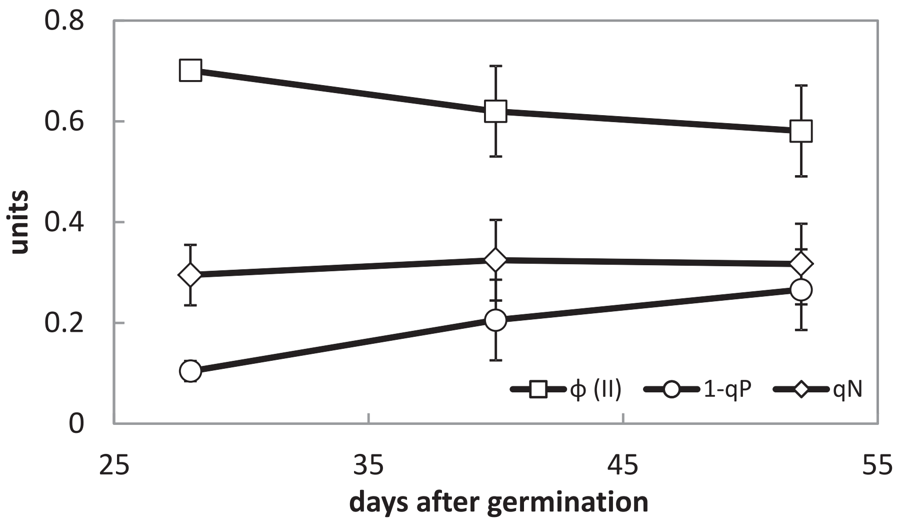
0.8
0.6
0.4
0.2
0
units
25
35
45
55
days after germination
ϕ (II)
1-qP
qN

Figure S3

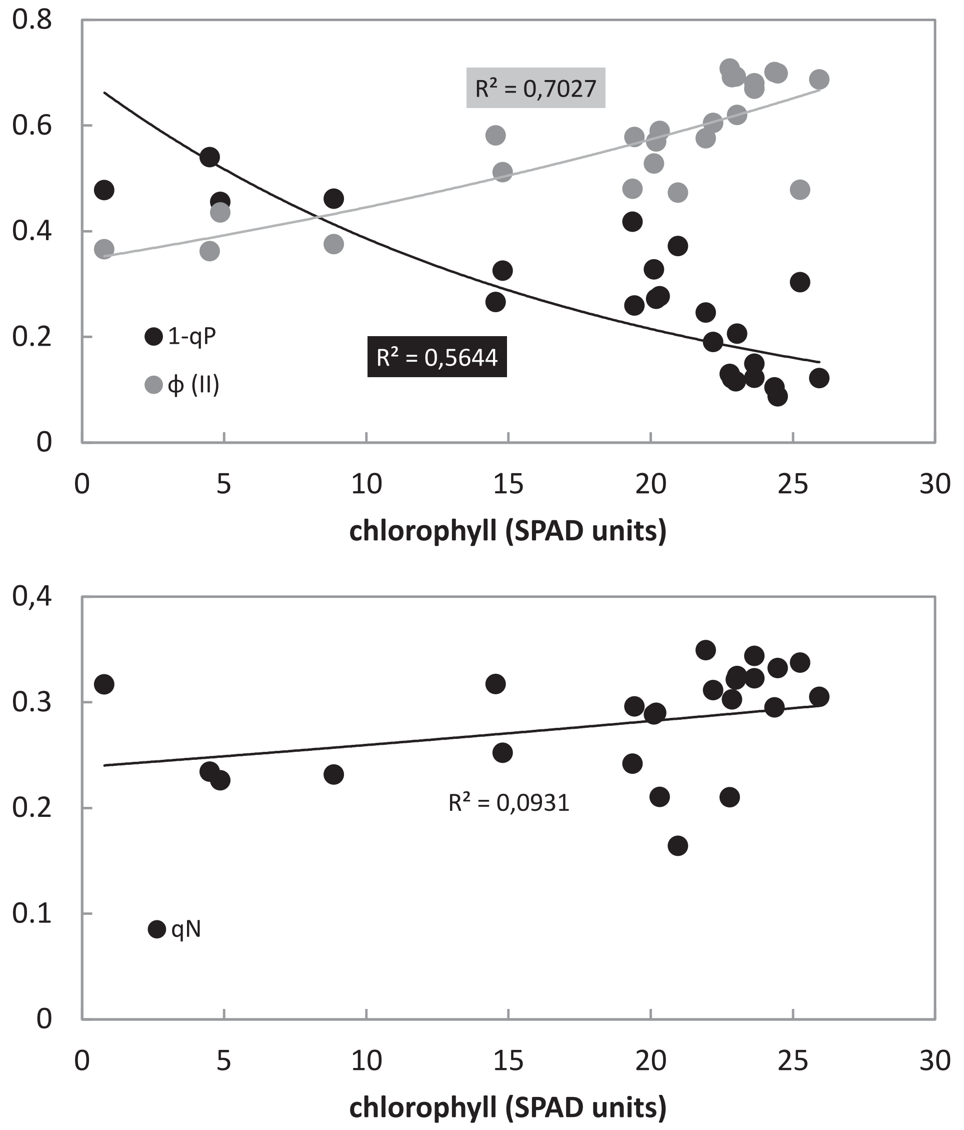
0.8
0.6
0.4
0.2
0
R² = 0,7027
R² = 0,5644
1-qP
ϕ (II)
0
5
10
15
20
25
30
chlorophyll (SPAD units)
0,4
0.3
0.2
0.1
0
R² = 0,0931
qN
0
5
10
15
20
25
30
chlorophyll (SPAD units)

Figure S4

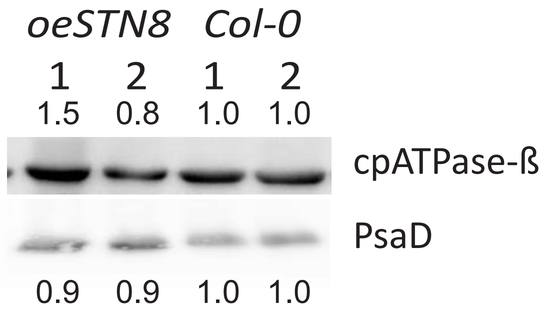

Figure S5

Supplementary Table 1. Photosynthetic efficiency measured as F_v/F_m, ϕ_{II}, 1-qP and qN in leaf No.6 of WT and mutants of *Arabidopsis thaliana* at the beginning of age-dependent senescence (day 28).

	F_v/F_m	ϕ_{II}	1-qP	qN
Col-0	0.84±0.01	0.70±0.02	0.10±0.02	0.30±0.06
psad2-1	0.84±0.01	0.69±0.04	0.12±0.02	0.32±0.09
psal-2	0.84±0.01	0.69±0.02	0.12±0.02	0.31±0.10
psan-2	0.84±0.01	0.69±0.04	0.12±0.03	0.30±0.08
psbs	0.85±0.00	0.71±0.03	0.13±0.03	0.21±0.02*
pete2-1	0.85±0.01	0.59±0.02*	0.28±0.02*	0.21±0.04*
stn7-1	0.84±0.01	0.68±0.02	0.12±0.01	0.29±0.04
stn8-1	0.83±0.02	0.70±0.02	0.09±0.04	0.28±0.07
stn7 stn8	0.83±0.01	0.62±0.04*	0.16±0.02*	0.30±0.05
oeSTN7	0.84±0.01	0.71±0.02	0.09±0.02	0.29±0.04
oeSTN8	0.84±0.00	0.70±0.01	0.12±0.01	0.31±0.02

Significant deviation from the WT plants ($p < 0.05$) is indicated by asterisk.

Supplementary Table 2. Photosynthetic efficiency measured as F_v/F_m, ϕ_{II}, 1-qP and qN in leaf No.6 of WT and mutants of *Arabidopsis thaliana* at day 40.

	F_v/F_m	ϕ_{II}	1-qP	qN
Col-0	0.84±0.02	0.62±0.09	0.21±0.08	0.32±0.08
psad2-1	0.83±0.01	0.60±0.09	0.19±0.07	0.31±0.09
psal-2	0.81±0.02	0.48±0.11*	0.30±0.06*	0.34±0.09
psan-2	0.84±0.02	0.57±0.09	0.27±0.10	0.29±0.07
psbs	0.77±0.13	0.47±0.14*	0.37±0.11*	0.16±0.04*
pete2-1	0.83±0.02	0.48±0.08*	0.42±0.09*	0.24±0.04*
stn7-1	0.83±0.02	0.61±0.09	0.25±0.09	0.33±0.08
stn8-1	0.83±0.02	0.61±0.13	0.26±0.16	0.29±0.04
stn7 stn8	0.83±0.04	0.58±0.12	0.28±0.13	0.29±0.08
oeSTN7	0.83±0.04	0.66±0.02	0.20±0.03	0.34±0.04
oeSTN8	0.83±0.00	0.56±0.09	0.23±0.11	0.50±0.05*

Significant deviation from the WT plants ($p < 0.05$) is indicated by asterisk.
